# EFFECT OF PHYSICAL EXERCISE ON COGNITION IN ADULT DAY PARTICIPANTS

**DOI:** 10.1093/geroni/igz038.2421

**Published:** 2019-11-08

**Authors:** Bradford Stucki, Ben Katz, Jonathan Briganti, Ila Schepisi, Shannon Jarrott

**Affiliations:** 1 Virginia Tech, Blacksburg, Virginia, United States; 2 The Ohio State University, Columbus, Ohio, United States

## Abstract

Aerobic exercise has been demonstrated to be one of the most effective means of improving cognition in older adults. However, less is known about how exercise programs may improve cognition in older adults participating in Adult Day Service (ADS) programs. We analyzed a ten-year longitudinal data set from the Virginia Tech ADS center. We limited our analyses to individuals for whom we had two time points of the Mini-Mental Status Exam (MMSE) (n=142; average age = 78.48; 63 female, average days at center = 347; SD=432.71). Participants in the center completed approximately 30 minutes of physical exercise each day of attendance. The exercise regimen was largely composed of aerobic chair exercise, stretching, and lifting. Facilitator ratings of engagement with the exercise activity between the two test administrations were used to create an average engagement score for each participant. Multiple regression analyses were conducted using engagement as a predictor and change in MMSE as an outcome; no significant relationship was identified between exercise engagement and change in cognitive status. However, a moderation analysis conducted with diagnosis of Alzheimer’s disease (AD) or dementia as a predictor, change in MMSE as an outcome, and exercise engagement as a moderator revealed a significant moderation effect (p = .001). Greater exercise engagement was associated with improvements on the MMSE, but only for individuals without a diagnosis of AD or dementia. Given that many ADS programs serve individuals both with and without AD or dementia, these findings may inform more personalized exercise interventions at ADS centers.

